# *Leishmania* in Texas: A Contemporary One Health Scoping Review of Vectors, Reservoirs, and Human Health

**DOI:** 10.3390/biology14080999

**Published:** 2025-08-05

**Authors:** Morgan H. Jibowu, Richard Chung, Nina L. Tang, Sarah Guo, Leigh-Anne Lawton, Brendan J. Sullivan, Dawn M. Wetzel, Sarah M. Gunter

**Affiliations:** 1 National School of Tropical Medicine, Baylor College of Medicine, Houston, TX 77030, USA; morgan.jibowu@bcm.edu (M.H.J.); nina.tang@bcm.edu (N.L.T.); 2 William T. Shearer Center for Human Immunobiology, Texas Children’s Hospital, Houston, TX 77030, USA; 3 Department of Kinesiology, Rice University, Houston, TX 77251, USA; rc89@rice.edu (R.C.); sg156@rice.edu (S.G.); 4 Texas Department of State Health Services, Region 6/5 South, Houston, TX 77023, USA; leighanne.lawton@dshs.texas.gov (L.-A.L.); brendan.sullivan@dshs.texas.gov (B.J.S.); 5 Department of Pediatrics, University of Texas Southwestern Medical Center, Dallas, TX 75390, USA; dawn.wetzel@utsouthwestern.edu; 6 Department of Biochemistry, University of Texas Southwestern Medical Center, Dallas, TX 75390, USA

**Keywords:** leishmaniasis, *Leishmania*, Texas, One Health, vectors, reservoirs, epidemiology, review

## Abstract

Leishmaniasis, a vector-borne neglected tropical disease, affects over 6.2 million people globally. Case acquisition is increasingly recognized in the United States, and in Texas, most reported cases are locally acquired and speciated to *Leishmania mexicana*. We conducted a scoping literature review to systematically assess contemporary research on *Leishmania* in humans, animals, reservoir hosts, or vectors in Texas after 2000. Out of 22 eligible studies, the most prevalent themes were case reports, followed by studies on domestic animals, reservoirs, and vectors, with several studies bridging multiple disciplines. Climate change, urbanization, and habitat encroachment appear to be driving the northward expansion of *L. mexicana*, which is primarily attributed to shifts in the habitats of key vectors (*Lutzomyia anthophora*) and reservoirs (*Neotoma* spp.). *Leishmania* appears to be expanding into new areas, with potential for further spread. As ecological conditions evolve, strengthening surveillance and clinician awareness is crucial to understanding disease risk and improving early detection and treatment in affected communities.

## Simple Summary

Leishmaniasis is a parasitic disease spread by sand flies that affects millions of people worldwide. Although it is typically found in tropical areas, cases are now being identified in the United States, particularly in Texas. In this region, the species *Leishmania mexicana* (which causes skin sores) is present. Most infections appear to be locally acquired, meaning that people become infected without traveling out of the state. This review brings together existing research on leishmaniasis in Texas, examining how the disease spreads, which animals may harbor the parasite, and the influence of the environment. We found that rising temperatures, expanding cities, and habitat changes may be contributing to the movement of the disease into new areas. These findings demonstrate the need for stronger disease tracking and increased awareness among healthcare providers to improve early diagnosis and treatment. A better understanding of how leishmaniasis is spreading in Texas will support efforts to protect communities and guide public health efforts.

## 1. Introduction

Leishmaniasis is a neglected tropical disease that disproportionately affects underserved and resource-limited communities worldwide [1]. It is caused by protozoan parasites of the *Leishmania* genus, which are transmitted primarily through the bites of infected female phlebotomine sand flies. The disease causes significant global health burdens, affecting over 6.2 million people, with an estimated 1.1 million new cases annually [2,3]. Among the three primary clinical forms of leishmaniasis—cutaneous, mucocutaneous, and visceral—cutaneous leishmaniasis (CL) is the most commonly reported, responsible for nearly 400,000 disability-adjusted life years (DALYs) [3].

While CL is traditionally associated with tropical and subtropical regions of South America, Central America, and parts of Mexico, domestically acquired cases are increasingly recognized in the United States. Autochthonous transmission has been documented in multiple states, with Texas emerging as a key region for understanding the dynamics of domestic transmission. Cases were first documented in Texas in 1903, and most reported CL cases in Texas have been locally acquired [4], highlighting the need for enhanced surveillance and targeted public health interventions.

Previous literature reviews on leishmaniasis have primarily focused on the epidemiology and transmission dynamics of *Leishmania* in highly endemic regions, such as South America and the Middle East [4]. The number of reviews assessing human case distribution, vector ecology, and environmental drivers of transmission within Texas and the United States remains limited. This information is essential for strengthening vector surveillance, improving case detection, and informing public health strategies.

To address this gap, we conducted a scoping literature review to systematically assess existing research on leishmaniasis in Texas and identify knowledge gaps. Specifically, we examine human cases, vector ecology, reservoir hosts, and environmental drivers influencing transmission in Texas. Using a “One Health” approach, we provide a holistic perspective on *Leishmania* in Texas to inform research needs, enhance surveillance efforts, and support disease mitigation strategies.

## 2. Materials and Methods

We followed the Preferred Reporting Items for Systematic Reviews and Meta-analysis Protocols Extension for Scoping Reviews (PRISMA-ScR) (Supplementary File S1).

### 2.1. Study Identification

We searched OVID Medline and PubMed for articles published between 2000 and 2024 using (“*Leishmania*” OR “leishmaniasis”) AND (“Texas” OR “TX”), applied as keywords, titles, abstracts, and Medical Subject Headings (MeSH) terms. This time frame was deliberately chosen to reflect contemporary (rather than historical) epidemiological patterns in Texas. We included studies published in English after 2000 that contained primary research examining *Leishmania* in humans, domestic or wild animals, reservoir hosts, or vectors within the state of Texas. Papers were excluded if they focused solely on laboratory experiments or were conference abstracts without full-text availability. The most recent search was performed in January 2024, and the complete search strategy is provided in Supplementary File S2. To capture the relevant gray literature, we searched ProQuest for theses and dissertations.

### 2.2. Study Screening

All search results were exported into reference management software (Zotero version 6.0.36), in which the duplicates were removed. Two reviewers screened all publications’ titles, abstracts, and full texts based on the eligibility criteria. Discrepancies were resolved through discussion and consultation with a third reviewer if needed. Additionally, we screened references of included studies to identify additional relevant literature.

### 2.3. Data Extraction and Synthesis

A structured data-charting form was developed to extract key variables, including article details (authors, journal, and publication year) and study characteristics (study location, objectives, methodology, sample size, and outcomes). Three reviewers collaboratively charted the data, discussed the results, and continuously updated the data-charting form in an iterative process.

### 2.4. Data Summarization and Presentation

**Figure 1:**
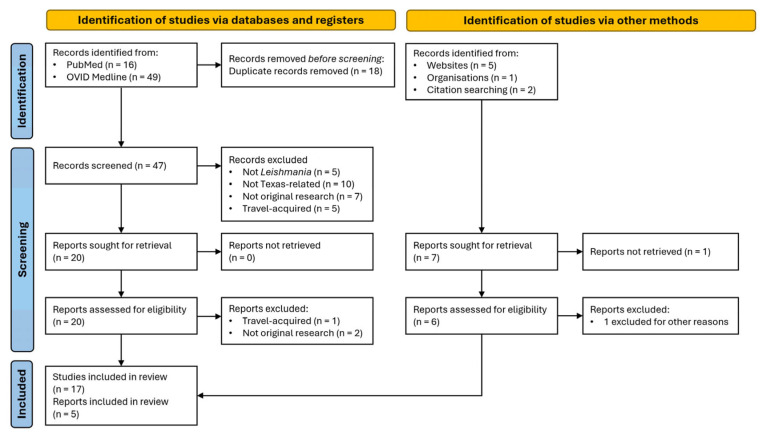
Study selection process using PRISMA-ScR guidelines. Flow diagram showing the identification, screening, eligibility assessment, and inclusion of studies in the scoping review of *Leishmania* in Texas.

Studies were categorized based on their outcomes, and included human cases, domestic animal infections, reservoir hosts, and/or vector surveillance. Summary statistics were used to describe the distribution of the literature across study themes, designs, and locations. The study selection process is shown in Figure 1.

## 3. Results

### 3.1. Study Selection

After the full-text screening, 22 articles (17 peer-reviewed and 5 gray literature reports) met the eligibility criteria and were included in this review. Table 1 summarizes the geographic focus, study period, study design, outcomes, primary findings, and classification (human, reservoir, domestic animal, and/or vector) of the included studies. The most prevalent themes were human disease (n = 7, 32%), followed by domestic animals (n = 4, 18%), reservoirs (n = 3, 14%), and vector studies (n = 3, 14%). Several studies have bridged disciplines, including some combination of human disease, domestic animal disease, vector, and reservoir studies (n = 5, 23%).

The studies used various methods and are broadly categorized into clinical studies or case reports (n = 7, 32%), epidemiological studies (n = 2, 9%), molecular diagnostics (n = 2, 9%), and ecological investigations (n = 11, 50%). Clinical studies included case reports, retrospective reviews, and evaluations based on patient presentation, biopsies, and histopathology. Epidemiological studies were primarily cross-sectional or retrospective case reviews, often incorporating assessments of travel history. Molecular approaches commonly included PCR and sequencing to detect and characterize *Leishmania* species. Ecological studies focused on cross-sectional or longitudinal sand fly and rodent trapping, species composition analysis, ecological niche modeling, and susceptibility testing. Several studies integrated multiple approaches to provide a more comprehensive understanding of transmission dynamics. There was a notable lack of research on prevention or treatment.

### 3.2. Human Cases, Clinical Features, Diagnosis, and Treatment

#### 3.2.1. Human Cases

Human leishmaniasis cases were first documented in Texas in 1903 near the southeastern border with Mexico [13,26]. By the mid-1940s, cases had been reported in south-central Texas, reaching central Texas by the early 1980s and spreading further north by the 1990s [10,13]. Recognizing its growing presence, Texas became the only U.S. state to designate leishmaniasis as a reportable condition in 2007, requiring healthcare providers to report suspected and confirmed cases to the Texas Department of State Health Services (DSHS) [20].

Over the past 25 years, autochthonous CL has been increasingly documented, with its range now extending from Texas into southeastern Oklahoma [20]. However, leishmaniasis in Texas is likely underreported, as the literature documents the discovery of previously unreported cases [4,24]. For example, a recent study of CL in Texas reported 69 human cases of CL since 2007, only 14 of which were reported to DSHS [4]. Despite increasing numbers of case reports, the actual burden of leishmaniasis is unclear, due to the lack of prevalence data in Texas.

In Texas, *Leishmania mexicana* is the predominant species identified in humans, animals, and vectors [5]. Multiple studies have conducted molecular characterization of *L. mexicana* isolates collected from human cases in Texas, detecting specific single-nucleotide polymorphisms (SNPs) in the RNA-Internal Transcribed Spacer-2 (ITS-2) gene [24]. Unique polymorphisms at ITS-2 C647 and C649 have been identified in Texas isolates, and non-travel-associated *L. mexicana* infections in Texas predominantly belong to genotype CCC [24,25]. Additional polymorphisms in genes such as mannose phosphate isomerase (MPI), malate dehydrogenase (MDH), and 6-phosphogluconate dehydrogenase (6PGD) have been described [24]. Additionally, interestingly, despite characterizing a limited number of isolates and genes, Nepal et al. observed genetic variations among strains, suggesting that *L. mexicana* strains in Texas may be experiencing evolutionary selection pressure leading to novel synonymous and nonsynonymous mutations [24]. Regardless, Texas-specific polymorphisms indicate ongoing enzootic disease transmission in Texas and potentially unique transmission dynamics. Further studies are needed to determine whether these polymorphisms influence clinical presentation, disease severity, and/or treatment response.

#### 3.2.2. Clinical Features

Since 2000, 58 unique cases of autochthonous cutaneous leishmaniasis (CL) have been reported in the literature in Texas residents (Table 2). The year of diagnosis was provided for 94.8% (55/58) of cases, and ranged from 2005 to 2019. Notably, the first half of this period (2005-2011) resulted in only 17 cases, whereas 38 cases were diagnosed between 2012 and 2019, suggesting an increasing incidence or awareness of autochthonous CL in Texas. In the 29.3% of cases with a reported age, the median age of autochthonous CL cases was 64 (range: 6 months - 82 years). With thirteen adult and four pediatric cases, there was an apparent skew towards older populations. This may reflect age-related healthcare-seeking behaviors, especially as CL lesions can mimic dermatologic neoplasms [19,21].

Autochthonous cases of CL spanned 23 Texas counties, with the most common counties being Collin (6), Dallas (6), Grayson (6), and Denton (5) (Table 2). Most commonly, lesions were found on the face and neck (n = 26) or upper extremity (n = 24). However, three cases included multiple lesions that spanned various anatomic locations. Most cases initially presented to primary care physicians or dermatologists, while one presented to an ophthalmologist as the lesion was located on the eyelid [14]. 

#### 3.2.3. Diagnosis and Treatment

The duration between lesion appearance and appropriate diagnosis was reported in 16 cases, with a median time to diagnosis of 4 months. All diagnoses were made using histopathology; however, initial punch biopsies were inconclusive in two cases [6,19]. This necessitated further excisions, which yielded the correct diagnosis of CL. Further speciation was performed in 43.1% of the evaluated cases, and *L. mexicana* was identified.

Since *L. mexicana* typically causes non-disseminating cutaneous disease, the Infectious Diseases Society of America and the American Society of Tropical Medicine and Hygiene advise that immunocompetent hosts do not require treatment [27]. However, treatment can be provided for cosmetic reasons, as non-treatment may be associated with superinfection or more pronounced scarring [27]. In the U.S., such treatments may include amphotericin B, antimonials, miltefosine, “azole” antifungal compounds, paromomycin, heat therapy, and cryotherapy [27]. In the nine cases that included information regarding clinical courses and treatments, a combination of pharmacologic and non-pharmacologic regimens was applied, including various treatment combinations. Overall, these patients were treated with amphotericin B, fluconazole, miltefosine, ketoconazole, paromomycin, heat therapy, cryotherapy, and/or lesion excision.

In addition to recommended anti-leishmanial management, six individuals received antibiotic treatment [10,14,19,24]. Four of these individuals were given antibiotics empirically before the diagnosis of CL was made [10,14,24], while one individual tested positive for *Proteus mirabilis* and *Klebsiella oxytoca* on cultures collected during the diagnostic biopsy [19]. In one case reported by Wright et al., gentamicin was used; however, it is unclear whether it was administered empirically prior to diagnosis [10]. Empiric treatment with corticosteroids was also observed in five cases. In Maloney et al., the initial punch biopsy showed findings that suggested nonspecific chronic inflammation, warranting treatment with topical steroids [6]. However, in the remaining four cases, corticosteroids were prescribed empirically (before the diagnosis was made) and reportedly worsened lesions in three cases [10,20].

Together, these cases suggest that autochthonous CL is a growing zoonotic threat to human health in Texas, where the incidence of reported autochthonous disease appears to have increased by 124%, based on a comparison of the period from 2012 to 2019 with the period from 2005 to 2011. As the U.S. historically has been declared non-endemic for leishmaniasis, increasing physician awareness of the potential for autochthonous CL in individuals with no significant travel history is crucial. Due to the observed skew of autochthonous CL reported in older individuals and the reported cases in which CL lesions have been misdiagnosed as dermatologic neoplasms, providers in diverse specialties, including primary care, family medicine, and dermatology, must consider CL in their differentials. This is likely to improve patient outcomes by reducing the time to diagnosis, the incidence of misdiagnoses, and the use of empiric antibiotics and corticosteroids that may ultimately exacerbate CL lesions. 

### 3.3. Sand Flies

Several studies have identified *Lutzomyia* species as potential vectors of *Leishmania* spp. in Texas. While approximately 70 distinct sand fly species worldwide have been identified as competent vectors for *Leishmania*, *Lutzomyia anthophora* is the only species definitively identified as a vector for *L. mexicana* transmission in Texas (Figure 1) [5,23,28]. This was reported by Kipp et al., who detected human blood in *Lu. anthophora* for the first time [20]. Although other sand fly species, including *Lu. aquilonia, Lu. diabolica, Lu. shannoni, Lu. texana*, and *Lu. vexator* have been collected in Texas; none have demonstrated a natural infection with *L. mexicana* [4,5,11,14,15,20,23]. Therefore, the ecology and vectorial competency of additional *Lutzomyia* species previously found in Texas require further investigation [11].

#### 3.3.1. Sand Fly Biology and Behavior

*Lu. anthophora* is active February–November, with periods of peak abundance and activity occurring in intervals of two months [5,29]. This species undergoes 3–5 generations annually, with synchronous egg hatching within 24 h [5]. Activity declines when mean monthly low temperatures fall below 10 °C, with most activity ending by September, although some collections have been reported as late as November [5]. Increased sand fly abundance has been associated with increased ambient air temperature and the temperature of woodrat nests [15], indicating that they may survive in these warmer environments during the winter. Year-round activity may be possible in southern Texas due to its warmer climate [15]. Data on the daily activity patterns of sand flies is limited, as traps are rarely set for an entire 24-h period. However, studies indicate peak activity at dusk, overnight, and dawn [11,14,15,23,24].

#### 3.3.2. Sand Fly Ecology and Distribution

Sand fly abundance and distribution in Texas appear to be shaped by ecological factors such as temperature, moisture, vegetation, and host availability. However, most existing studies rely on convenience sampling rather than systematic sampling, which limits our understanding of broader patterns. High sand fly abundance has been documented in shrubland, wetland, and forest habitats, and tends to correlate with domestic animal activity [23] and proximity to water [15]. Sand flies have also been collected in grasslands and woodland/scrub ecosystems [11]. Suburban and rural development encroaching on undeveloped lands may increase the risk for human exposure due to proximity to wildlife reservoirs such as burrowing woodrats, opossums, armadillos, and cotton rats [10]. Some evidence suggests that environmental modifications such as brush clearing and reducing outdoor lighting may help suppress local sand fly populations [23]. Although female sand flies primarily feed on rodents and other mammals, they may opportunistically bite humans and domestic animals, especially when preferred hosts are scarce [14]. Furthermore, their dispersal range and the effective range of light traps remain poorly understood. However, existing research suggests they are weak fliers and that transmission is typically localized around host habitats [5].

### 3.4. Roles of Wildlife and Domestic Animals

#### 3.4.1. Wildlife Reservoirs

**Figure 2:**
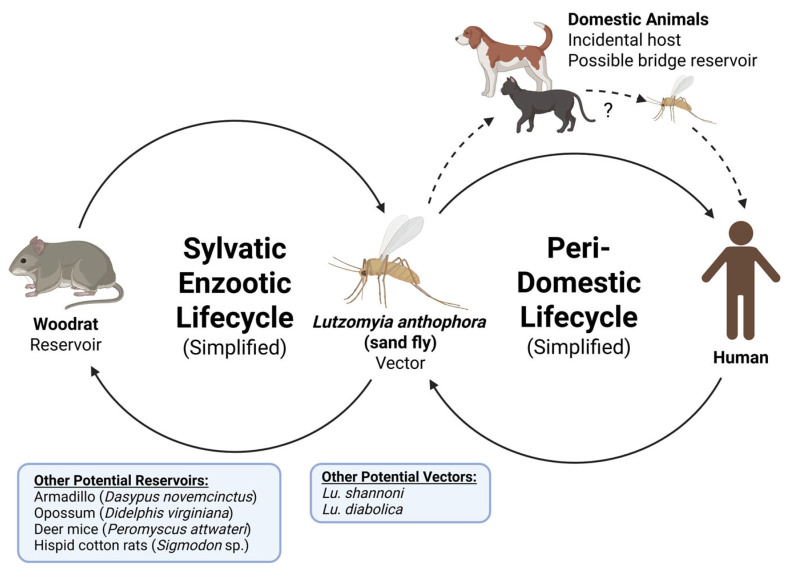
The proposed lifecycle for *Leishmania* in Texas.

Rodents, particularly woodrats (*Neotoma floridana* and *Neotoma micropus*), appear to be the primary reservoirs of *Leishmania* in Texas (Figure 1) [9]. *N. micropus* is abundant in the brushlands of southern Texas, with ecological ranges extending into New Mexico, Colorado, and portions of Kansas and Oklahoma [7]. Notably, infections in *N. micropus* have been reported to persist for an average of 190 days [9]. Interestingly, a case in Brenham, Texas, reported in 2002, was outside *N. micropus*’s range, suggesting an alternative reservoir in East Texas [6]. This is further supported by the discovery of an infected *N. floridana* 80 km away in a similar ecological region [9]. *Leishmania* has been detected in *Peromyscus attwateri* [18], while studies from northeastern Mexico implicate deer mice (*Peromyscus*) and hispid cotton rats (*Sigmodon*) [30]. Significantly, many of these ecological niches extend into the southern U.S., suggesting a broader geographic risk [4,7,13]. This emerging data suggests that additional rodent species may contribute to transmission (Figure 2), but further investigation is required.

#### 3.4.2. Domestic Animals

Stray canines and felines infected with *L. mexicana* have been documented across northern, central, and western Texas [12,17,18,23], with a notable study detecting *Leishmania* DNA in 41 stray dogs (26%) in El Paso County [17]. A study of stray cats in El Paso revealed that 19 (12%) tested positive for *L. mexicana* [16]. Infections in stray cats and dogs provide evidence of *L. mexicana* enzootic transmission in Texas, though studies suggest that they are incidental hosts rather than true reservoirs [17,18]. However, dogs and cats may act as “bridge reservoirs,” facilitating the movement of *Leishmania* from sylvatic to peridomestic settings (Figure 2) [22,31]. Therefore, further investigation is needed to understand their roles as potential reservoirs and any impact on human transmission.

### 3.5. Trends and Patterns

**Figure 3:**
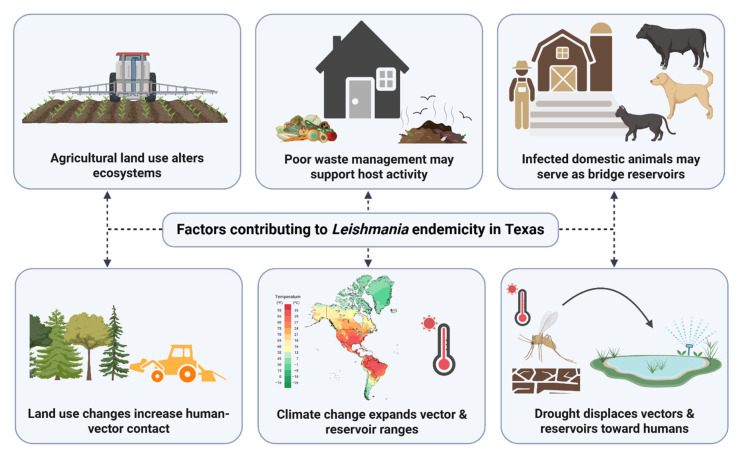
Potential factors contributing to changes in *Leishmania* endemicity in Texas.

Recent studies suggest that climate change, urbanization, and habitat encroachment reshape the distribution of *Leishmania* vectors and reservoirs in Texas (Figure 3) [4,13,17,32]. The northward expansion of *L. mexicana* is primarily attributed to shifts in the habitats of key vectors (*Lu. anthophora)* and reservoirs (*N. micropus* and *N. floridana*) [10,12,16]. For example, shifts in *N. micropus’* range to northern, wooded environments and changes in sand fly habitats may contribute to the northern spread of *L. mexicana* [10,12]. Other potential hosts, such as opossums, armadillos, and cotton rats, may facilitate transmission by sustaining necessary vector–host relationships.

Agricultural changes, including the spread of rodent crop pests (e.g., *Sigmodon* sp.), may also influence the distribution of *L. mexicana* [30]. However, some argue that urban expansion, habitat encroachment, and increased disease awareness may explain the rise in reported leishmaniasis cases, as opposed to an explanation based on actual geographic spread [10,19,20]. Regardless, climate models predict that the risk of Leishmania exposure could extend to southeastern Canada by 2050, underscoring the urgency of understanding how climate and human-modified environments may influence disease dynamics [4].

## 4. Discussion

The emergence and geographic expansion of *Leishmania mexicana* in Texas and the southern U.S. is a significant yet underrecognized public health concern. Once considered only travel-associated, cutaneous leishmaniasis is increasingly reported among individuals in the U.S. with no travel history [4]. These reports highlight the need to reframe cutaneous leishmaniasis as a locally acquired zoonosis in Texas and other U.S. regions in which autochthonous cases have been seen.

The presence of *Leishmania* in Texas reflects a complex ecology involving sylvatic vectors, rodent reservoirs, and environmental conditions conducive to transmission. *Lu. anthophora* is believed to be the primary vector, while woodrats and other rodents may sustain sylvatic enzootic cycles [5,9,23,28]. However, increasing human encroachment, land-use changes, and environmental stressors such as drought may facilitate spillover into peri-domestic and suburban settings by bringing humans and domestic animals closer to infected vectors and reservoirs [14]. Even in areas without rapid urbanization, stable environments with certain features, such as woodrat habitats and domestic fowl, may support persistent enzootic transmission [14]. Historical case reports illustrate how poor waste management, such as debris piles, abandoned vehicles, and nearby animal hosts, can sustain sand fly populations [14].

Despite its growing relevance, leishmaniasis remains poorly recognized in clinical settings within the United States. Physicians are responsible for identifying, diagnosing, and treating *L. mexicana* while correctly reporting it to public health authorities [4]. However, leishmaniasis is often viewed as an “exotic” or exclusively travel-associated disease, leading to low levels of physician awareness and frequent misdiagnosis in clinical settings [19,33]. Underreporting remains a significant challenge, especially outside Texas, where no public health reporting requirement exists. Enhanced surveillance systems for vectors, reservoirs, and human cases, improved diagnostic tools, and increased education for healthcare providers are essential to enhance disease monitoring and improve patient outcomes. Treatment for *L. mexicana* is often not indicated for immunocompetent adults with a single, small lesion, particularly if it is not in a cosmetically sensitive area. Nevertheless, most providers in the previously reported cases have often treated children and adults with a variety of methods, often because the case specifics varied from the above criteria. Some of these treatments have the potential for significant side effects, and there is also concern that *Leishmania* may develop resistance over time [34,35]. Of note, long-term CL sequelae include scarring, which may be decreased with treatment but is not eliminated. Since scarring—especially in more visible areas such as the face—can have significant impacts on individual mental health and social stigma, additional research on scar-mitigating treatment modalities on active and healed CL lesions would significantly benefit human health.

### 4.1. Research Gaps

Multiple knowledge gaps exist in our understanding of *L. mexicana* in Texas and the United States. First, the role of domestic animals in the *L. mexicana* transmission cycle is poorly understood. Whether stray canine and feline infections are incidental hosts or potential reservoirs contributing to sustained transmission is unclear [17,18]. Second, studies are needed to systematically map the parasite’s geographic range in Texas and evaluate the risk of human infection [20]. The northward expansion of reservoir and vector ranges, which is especially due to climate change, suggests that endemic *Leishmania* transmission may extend farther into the United States [13]. Ongoing surveillance is also needed to track potential northward expansion [10]. Genomic surveillance offers a promising avenue to detect strain-specific SNPs that may indicate ongoing transmission cycles in Texas that are distinct from those in Mexico and Central and South America. To assess genetic and immunological factors, comparisons of *L. mexicana* strains could determine how genetic variations influence clinical presentation, disease progression, and treatment response [20,24]. There is also a need to clarify the roles of lesser-known vectors, such as other *Lutzomyia* species and non-woodrat rodents like *Peromyscus attwateri*, in maintaining enzootic transmission [11,18]. Addressing these research gaps will provide a clearer understanding of the epidemiology of *L. mexicana* and guide strategies to mitigate its impact on human and animal health.

### 4.2. Vector Control Strategies

Leishmaniasis prevention continues to rely heavily on vector control, including environmental management and insecticide use [32]. Primary insecticide methods include indoor residual spraying, insecticide-treated nets, repellants, and impregnated dog collars. However, overreliance on these tools can lead to insecticide resistance [36]. Local and state vector control programs are not equipped to conduct sand fly surveillance or test sand fly populations for *Leishmania* spp. Encouragingly, surveillance could be adapted into existing vector control programs, as sand flies can be found in bycatch during mosquito trapping. In addition, oral insecticides from the isoxazoline class (e.g., fluralaner, sold as Bravecto®) have demonstrated efficiency against specific sand fly species [37,38,39]. Further research is needed to determine whether these effects extend to the sand fly species found in Texas. This approach could represent a valuable One Health intervention if domestic dogs and cars serve as bridge reservoirs.

### 4.3. Implications for Policy and Practice

Several changes are needed to better prevent and control leishmaniasis in the United States. Adding leishmaniasis to the list of nationally notifiable conditions would enable a more consistent surveillance and detection of outbreaks. In addition, improved diagnostic tools and provider education are essential to reduce misdiagnosis and enhance case detection. Early disease recognition is important given that *L. mexicana* infection can cause prominent scarring and even mucosal or visceral leishmaniasis in immunocompromised patients [27,40]. Lastly, public health authorities should encourage diagnosis and reporting of cases and integrate One Health approaches into prevention efforts. These steps will help clarify the disease burden and guide evidence-based responses.

### 4.4. Strengths and Limitations

The primary strength of this review lies in its comprehensive identification of the English-language literature related to leishmaniasis in Texas, including gray literature reports. We used an extensive and systematic search strategy with collaboration from a multidisciplinary team. This approach allowed us to assess studies on vectors, reservoirs, and domestic animals and provide a holistic One Health perspective on the disease in Texas. However, we acknowledge that our study has several limitations. We excluded studies published before 2000 in order to focus on the current ecological context, which may have excluded some relevant contemporary data. We also limited the inclusion criteria to articles explicitly discussing leishmaniasis in Texas, though this could have excluded broader studies with relevant implications. In addition, we developed the study classifications after reading the full texts, which could have introduced bias; however, we believed this was necessary for a comprehensive assessment. Finally, we excluded travel-acquired cases, though some could have been misclassified, with infections potentially occurring before departure or after return to Texas.

## 5. Conclusions

Leishmaniasis was first identified in southern Texas in 1903 and has since expanded into Oklahoma. The actual disease burden is likely underestimated due to underdiagnosis and underreporting. Although ecological data and surveillance are limited, current evidence suggests an increasing disease burden and continued geographic spread. Contributing factors include human encroachment into endemic areas and climate change, which may alter the distribution of vectors and reservoirs.

Effectively addressing *Leishmania* in Texas will require a One Health approach integrating human, animal, and environmental health perspectives. Furthermore, strengthening surveillance infrastructure and increasing clinician awareness are crucial for informing public health efforts and understanding the disease risk in our communities. Ultimately, reframing *L. mexicana* as a locally relevant zoonosis and responding with multidisciplinary One Health strategies will improve recognition, guide prevention and control strategies, and reduce the disease burden in affected communities.

## Figures and Tables

**Table 1:** Characteristics of included manuscripts.

First Author (Year)	Main Objective	Study Period	Geographic Focus	Methods and Outcome Measures	Main Findings	Category for This Review	Reference
McHugh (2001)	To describe sand fly abundance and biology at two human leishmaniasis foci	1997–1999	Medina County, Texas; Lackland Air Force Base, Bexar County, Texas	Longitudinal sand fly collection, species composition, and abundance analysis	*Lutzomyia anthophora* was the predominant species. *Lutozymia diabolica* and *Lutozymia texana* were also collected. One blood-fed *Lu. anthophora* was *Leishmania*-positive.	Vector	[5]
Maloney (2002)	To present a locally acquired CL case diagnosed by electron microscopy	Unknown	Brenham, Washington County, Texas	Case report; used electron microscopy to identify *Leishmania* spp. in tissue samples	Confirmed CL diagnosis in a Texas patient	Clinical – case report	[6]
Merkelz (2002)	To study the population dynamics of the Southern Plains woodrat (*Neotoma micropus*) and assess the prevalence of *L. mexicana*	1997–1998	La Copita Research Area, Jim Wells County, Texas	Longitudinal trap, release, and recapture; quarterly PCR on ear punch biopsies	No *Leishmania*-positive woodrats. Year-round breeding observed.	Reservoir	[7]
Raymond (2003)	To determine spatial and seasonal variations in *Leishmania* prevalence in Southern Plains woodrats (*N. micropus*)	1998–2000	Lackland Air Force Base, Bexar County, Texas	Longitudinal trapping, PCR on ear biopsies	A 14.7% prevalence of *L. mexicana*. Of the incident cases for which a transmission period could be estimated, most appeared to have acquired their infections during the year’s cooler months. Mean persistence of 191 days.	Reservoir	[8]
McHugh (2003)	To report disseminated *L. mexicana* infection in an eastern woodrat (*Neotoma floridana*)	Jan 2001	Bedias, Grimes County, Texas	Cross-sectional; Biopsy, PCR, clinical presentation	*L. mexicana* was detected in both ears and all feet, demonstrating disseminated cutaneous infection. Oral and nasal mucosa were negative.	Reservoir—case report	[9]
Wright (2008)	To report the first autochthonous CL cases in North Texas	2005–2007	Dallas–-Fort Worth, Texas	Cross-sectional Histological examination	Nine cases, all Caucasian, even sex distribution. No travel reported.	Clinical—case reports	[10]
Claborn (2009)	To conduct surveillance of sand fly populations and their susceptibility to Old World *Leishmania* sp.	Late spring and summer 2006–2007	Fort Hood, Bell County, Texas; Fort Bragg, North Carolina; Fort Campbell, Kentucky	Longitudinal Sand fly collection, species diversity, and susceptibility testing	Identified five sand fly species (*Lutozymia shannoni, Lutozymia aquilonia, Lutozymia vexator, Lu. diabolica,* and *Lu. anthophora*). *Lu. shannoni* was susceptible to *Leishmania major*.	Vector	[11]
Trainor (2010)	To report autochthonous *L. mexicana* infections in eight domestic cats	2004–2008	Bastrop, Bell, Burleson, Caldwell, Hood, Kaufman, Lampasas, Tarrant Counties, Texas	Cross-sectional Skin biopsy, histological examination, and PCR	Amastigotes visualized. In total, 5/8 cats PCR-positive for *L. mexicana/Leishmania amazonensis*.	Domestic animal	[12]
Gonzalez (2010)	To assess potential climate change impacts on sand fly vectors (*Lu. anthophora* and *Lu. diabolica*) and reservoir species (*N. albigula, N. micropus, N. floridana, N. mexicana*)	Post–1990	United States, Mexico, and Canada	Ecological niche model using Maxent (presence/absence)	There is a north–south band of habitat that is, at best, marginally suitable for any of the four *Neotoma* species models. This may explain the temporal pattern of the spread of *Leishmania* cases in Texas. The pre-2000 records of leishmaniasis from Texas fall within the area predicted to be a suitable habitat for *Neotoma micropus*. Identified potential shifts in *N. floridana* and *Lu. diabolica* in eastern North America and *N. micropus* and *Lu. anthophora* further west.	Modeling; Reservoir; Vector	[13]
Clarke (2013)	To report three autochthonous CL cases and the collection of rodents and sand flies near two case-patients	2003–2006	Lamar County, Texas; Collin County, Texas; McCurtain County, Oklahoma (n = 2)	Cross-sectional Case reports, sand fly and rodent trapping	One case was rural, and one case was an urban-rural interface. No *Leishmania*-positive woodrats. Two species of sand flies were identified (*Lu. anthophora* and *Lu. vexator*).	Clinical—case reports; Reservoir; Vector	[14]
Alshhrany (2016)	To investigate seasonal sand fly abundance and related environmental factors	2014–2015	Poth, Texas; Wilson County, Texas	Longitudinal Sand fly collection, woodrat nest, and ambient temperature recording.	Identified two sand fly species (*Lu. anthophora, Lu. texana*). Correlation between sand flies and maximum ambient/nest temperatures, but not minimum ambient temperature.	Vector	[15]
Gonzalez (2015)	To determine the *Leishmania* spp. prevalence among stray cats collected in El Paso County, Texas	2014–2015	El Paso County, Texas	Cross-sectional Visualized for skin lesions and organ discoloration, biopsy, and PCR	In total, 12% of stray cats were infected with *Leishmania mexicana*. All cases occurred in July–December 2014. Of positives, 47% lacked skin lesions and organ discoloration.	Domestic animal	[16]
Kipp (2015)	To determine the *Leishmania* spp. prevalence among stray dogs collected in El Paso County, Texas	2014–2015	El Paso County, Texas	Cross-sectional Visualized for skin lesions and organ discoloration, biopsy, and PCR	In total, 26% of stray dogs tested positive for *Leishmania mexicana*. 71% of positive samples lacked visible skin lesions.	Domestic animal	[17]
Kipp (2016)	To screen stray dogs and sylvatic mammals for *Leishmania* spp.	2011–2012	El Paso County, Texas Mason County, Texas	Cross-sectional Visually examined for the presence of skin lesions; skin biopsy, and PCR	One *L. mexicana*-positive stray dog from a peri-urban, agricultural area adjacent to El Paso. One mouse, *Peromyscus attwateri,* was positive for *L. mexicana.* Stray dog infection is likely incidental.	Reservoir; Domestic animal	[18]
Oetken (2017)	To report an autochthonous CL case misdiagnosed as squamous cell cancer	2014	Cuero, Texas; DeWitt County, Texas	Cross-sectional Clinical presentation and histology	PCR-confirmed *L. mexicana.*	Clinical—case report	[19]
McIlwee (2018)	To assess the endemicity of human leishmaniasis in the United States using a multicenter observational study	2007–2017	Various counties throughout northern and central Texas	Retrospective case review of diagnosed leishmaniasis cases Determined travel history for the last 10 years	All autochthonous cases were from Texas. Of these, only 20% were reported to the Texas Department of State Health Services (DSHS). A total of 32% were speciated by PCR as *L. mexicana.*	Clinical—epidemiology	[4]
Kipp (2020)	To report an atypical, autochthonous CL case and sand fly collection near the case-patient’s residence	2016	Caldwell County, Texas	Punch biopsy, histopathology, sand fly collection	*L. mexicana* genotype C647/C640 identified. Six blood-fed *Lu. anthophora* contained human DNA. Of female sand flies, 3% were positive, and 1 had an identical sequence to the clinical case.	Clinical—case report; Vector	[20]
Nguyen (2020)	To describe an autochthonous CL case in North Texas	Unknown	Presented in North Texas	Skin biopsy, histopathology	Bar-shaped kinetoplast visualized. Received a curative single session of cryotherapy.	Clinical—case report	[21]
Meyers (2021)	To determine the prevalence of infection and exposure to vector-borne pathogens among working dogs	2015–2018	“Texas, where sand fly vectors occur”	Tested using Kalazar Detect Rapid Test Canine and PCR	A total of 4% of dogs were positive on the Kalazar Detect Rapid Test Canine, with two considered to have primary *T. cruzi*. None were positive by PCR.	Domestic animal	[22]
Hopke (2021)	To describe feline CL/mucocutaneous and report sand fly surveillance at the residence	Unknown	Bryan, Texas	Biopsy, PCR; Sand fly collection	*L. mexicana was* identified in both cutaneous and nasal mucosa samples. Identified *Lu. shannoni* (2) and *Lu. anthophora* (1) sand flies, all negative for *Leishmania*.	Domestic animal; Vector	[23]
Nepal (2024)	To report three CL cases in pediatric patients in northern Texas and propose a method for strain-typing US-endemic *L. mexicana*	2018–2019	Ellison County, Texas; Grayson County, Texas	Biopsy, multilocus sequence analysis (MLSA) for SNP analysis.	PCR confirmed *L. mexicana* infection. Clinical isolates exhibited genetic polymorphisms previously documented in Texan strains of *L. mexicana.*	Clinical—case reports; Clinical—diagnostics	[24]
de Almeida (2024)	To assess the prevalence of CL among travel- and non-travel-associated cases	2005–2019	United States	DNA sequencing of the ITS2 locus, genotyping of SNPs	In total, 90% of samples with the *L. mexicana* genotype CCC were from non-travelers identified in Texas	Clinical—epidemiology; Clinical—diagnostics	[25]

Abbreviations: CL = Cutaneous Leishmaniasis

**Table 2:** Clinical features of reported human cutaneous leishmaniasis cases in Texas.

Article Reporting	Age (Years)	Sex	Texas County of Origin	Ethnicity	Anatomic Location of Lesion (Specific)	Anatomic Location of Lesion (General)	Year of Histologic Diagnosis	Duration Before Diagnosis (Months)	Treatment *	Travel History (Last 5 Years)	Infective Species	Outcome	Reference
Wright et al., 2008	70	M	Dallas	C	R arm	upper extremity	2005	3	Amphotericin, fluconazole	None outside the county of residence	NR	NR	[10]
Clarke et al., 2013	74	F	Lamar	NR	L eyelid	face/neck	2005	1.5	Heat therapy	NR	NR	NR	[14]
Wright et al., 2008	60	F	Collin	C	Nose	face/neck	2006	1.5	Ketoconazole, cryotherapy	None outside the county of residence	NR	NR	[10]
Clarke et al., 2013 *	8	F	Collin	C	Face, L upper arm	multiple	2006	5	Amphotericin B, fluconazole	None outside the county of residence	*L. mexicana*	NR	[14]
Wright et al., 2008	76	F	Collin	C	L forehead	face/neck	2006	4	Cryotherapy	None outside the county of residence	NR	NR	[10]
Wright et al., 2008	64	M	Denton	C	R abdomen	trunk	2006	3	None	None outside the county of residence	NR	NR	[10]
Wright et al., 2008	80	M	Hill	C	L arm	upper extremity	2006	24	None	None outside the county of residence	NR	NR	[10]
McIlwee et al., 2018	NR	M	Wise	NR	R abdomen	trunk	2006	NR	NR	NR	NR	NR	[4]
Wright et al., 2008	57	M	Ellis	C	L back	trunk	2007	2	Fluconazole, surgical excision	NR	NR	NR	[10]
Wright et al., 2008	64	F	Ellis	C	R upper aspect of the chest	trunk	2007	1	Local excision, heat therapy	NR	NR	NR	[10]
McIlwee et al., 2018	NR	F	Grayson	C	Forearm, wrist	upper extremity	2007	NR	NR	NR	*L. mexicana*	NR	[4]
McIlwee et al., 2018	NR	F	Hill	C	Upper arm	upper extremity	2007	NR	NR	NR	*L. mexicana*	NR	[4]
Wright et al., 2008	82	F	Tarrant	C	L cheek	face/neck	2007	3	Fluconazole	NR	NR	NR	[4]
McIlwee et al., 2018	NR	F	Travis	NR	Chin, neck	face/neck	2007	NR	NR	NR	NR	NR	[4]
McIlwee et al., 2018	NR	M	Parker	NR	L wrist	upper extremity	2010	NR	NR	NR	NR	NR	[4]
McIlwee et al., 2018	NR	F	Dallas	C	Face, L elbow, buttock	multiple	2011	NR	NR	NR	*L. mexicana*	NR	[4]
McIlwee et al., 2018	NR	F	Rockwall	C	Upper arm	upper extremity	2011	NR	NR	NR	NR	NR	[4]
McIlwee et al., 2018	NR	M	Rockwall	C	Face	face/neck	2012	NR	NR	NR	*L. mexicana*	NR	[4]
McIlwee et al., 2018	NR	F	Brazos	NR	Forehead	face/neck	2013	NR	NR	NR	NR	NR	[4]
McIlwee et al., 2018	NR	F	Burleson	C	Upper shoulder	upper extremity	2013	NR	NR	NR	*L. mexicana*	NR	[4]
McIlwee et al., 2018	NR	M	Collin	NR	L arm	upper extremity	2013	NR	NR	NR	NR	NR	[4]
McIlwee et al., 2018	NR	F	Dallas	NR	R forehead	face/neck	2013	NR	NR	NR	*L. mexicana*	NR	[4]
McIlwee et al., 2018	NR	F	Dallas	C	Shoulder	upper extremity	2013	NR	NR	NR	NR	NR	[4]
McIlwee et al., 2018	NR	F	Denton	C	Upper arm	upper extremity	2013	NR	NR	NR	NR	NR	[4]
McIlwee et al., 2018	NR	M	Denton	C	Forearm	upper extremity	2013	NR	NR	NR	NR	NR	[4]
McIlwee et al., 2018	NR	F	Fayette	C	L eyelid	face/neck	2013	NR	NR	NR	NR	NR	[4]
McIlwee et al., 2018	NR	F	Grayson	NR	R lower eyelid	face/neck	2013	NR	NR	NR	NR	NR	[4]
McIlwee et al., 2018	NR	F	Hunt	C	R wrist	upper extremity	2013	NR	NR	NR	*L. mexicana*	NR	[4]
McIlwee et al., 2018	NR	F	Travis	C	Upper arm	upper extremity	2013	NR	NR	None outside the U.S.	*L. mexicana*	NR	[4]
McIlwee et al., 2018	NR	M	Bexar	NR	Upper eyelid	face/neck	2014	NR	NR	NR	*L. mexicana*	NR	[4]
McIlwee et al., 2018	NR	F	Caldwell	C	Face	face/neck	2014	NR	NR	NR	*L. mexicana*	NR	[4]
McIlwee et al., 2018	NR	F	Caldwell	NR	L cheek	face/neck	2014	NR	NR	NR	*L. mexicana*	NR	[4]
McIlwee et al., 2018	NR	F	Grayson	C	Face, eyelid	face/neck	2014	NR	NR	NR	NR	NR	[4]
McIlwee et al., 2018	NR	F	Grayson	NR	R forehead	face/neck	2014	NR	NR	NR	NR	NR	[4]
McIlwee et al., 2018	NR	F	Grayson	NR	L upper arm	upper extremity	2014	NR	NR	NR	NR	NR	[4]
McIlwee et al., 2018	NR	F	Madison	C	L temple	face/neck	2014	NR	NR	NR	*L. mexicana*	NR	[4]
McIlwee et al., 2018	NR	M	Tarrant	C	R ear	face/neck	2014	NR	NR	NR	*L. mexicana*	NR	[4]
McIlwee et al., 2018	NR	M	Wise	C	Forearm, a large portion of the arm	upper extremity	2014	NR	NR	NR	*L. mexicana*	NR	[4]
McIlwee et al., 2018	NR	F	Burleson	NR	L earlobe	face/neck	2015	NR	NR	NR	NR	NR	[4]
McIlwee et al., 2018	NR	F	Collin	C	Face	face/neck	2015	NR	NR	NR	*Leishmania* species	NR	[4]
McIlwee et al., 2018	NR	M	Collin	C	Ear, back	multiple	2015	NR	NR	NR	*L. mexicana*	NR	[4]
McIlwee et al., 2018	NR	M	Dallas	C	L forearm	upper extremity	2015	NR	NR	NR	NR	NR	[4]
McIlwee et al., 2018	NR	F	Denton	C	Forearm	upper extremity	2015	NR	NR	NR	*L. mexicana*	NR	[4]
McIlwee et al., 2018	NR	M	DeWitt	C	Face, cheek	face/neck	2015	NR	NR	NR	*L. mexicana*	NR	[4]
McIlwee et al., 2018	NR	F	Rockwall	NR	L upper arm	upper extremity	2015	NR	NR	NR	NR	NR	[4]
McIlwee et al., 2018	NR	M	Travis	C	Elbows	upper extremity	2015	NR	NR	NR	*Leishmania* species	NR	[4]
McIlwee et al., 2018	NR	F	Washington	NR	R dorsal hand	upper extremity	2015	NR	NR	NR	NR	NR	[4]
McIlwee et al., 2018	NR	F	Austin	NR	R anterior upper neck	face/neck	2016	NR	NR	NR	NR	NR	[4]
Kipp et al., 2020	67	M	Caldwell	C	R leg	lower extremity	2016	6	Fluconazole, miltefosine, ketoconazole	None outside Texas	*L. mexicana*	Treatment refractory 26 months later	[20]
McIlwee et al., 2018	NR	F	Dallas	NR	R face	face/neck	2016	NR	NR	NR	NR	NR	[4]
McIlwee et al., 2018	NR	M	Denton	NR	R upper arm	upper extremity	2016	NR	NR	NR	*L. mexicana*	NR	[4]
McIlwee et al., 2018	NR	F	Palo Pinto	NR	L forehead	face/neck	2016	NR	NR	NR	*L. mexicana*	NR	[4]
Nepal et al., 2024	2	F	Ellis	Hispanic	R inferior jaw	face/neck	2018-2019	6	Fluconazole	None outside North Texas	*L. mexicana*	Resolved	[24]
Nepal et al., 2024	3	M	Ellis	Hispanic	L arm	upper extremity	2018-2019	5	Fluconazole	None	*L. mexicana*	Resolved	[24]
Nepal et al., 2024	0.5	M	Grayson	C	R temple	face/neck	2018-2019	4	Fluconazole, paromomycin	None outside the U.S.	*L. mexicana*	Resolved	[24]
Oetken et al.	41	M	DeWitt	NR	L cheek	face/neck	NR	4	None	None to “endemic” areas	*L. mexicana*	Resolved	[19]
Maloney et al., 2002	78	F	Washington	NR	R forearm	upper extremity	NR	8	None	None outside Texas	Presumed *L. mexicana*	Improving without treatment	[6]
Nguyen et al	65	M	NR	NR	L shoulder	upper extremity	NR	multiple	Cryotherapy	None outside Texas	NR	Resolved	[21]

Abbreviations: F = Female; M = Male; NR = Not Reported; C = Caucasian; L = Left; R = Right; U.S. = United States. * Treatments do not include empirical treatments that were given before the diagnosis was made.

## Data Availability

Data sharing is not applicable to this article, as no datasets were generated or analyzed during the current study. Figures were created in BioRender.com.

## Supplementary Materials

The following supporting information can be downloaded at: https://www.mdpi.com/article/10.3390/biology14080999/s1, File S1: PRISMA ScR Checklist [41]; File S2: PubMed Search Strategy.
