# Supplementary material for: Leishmania in Texas: A Contemporary One Health Scoping Review of Vectors, Reservoirs, and Human Health
Source: Biology (Basel). 2025 Aug 5;14(8):999. doi: 10.3390/biology14080999 (PMC12383855; doi:10.3390/biology14080999)
Supplement: Supplementary file 1 [file biology-14-00999-s001.zip › Supplementary File S2_Search Strategy.pdf]

Jibowu, M.H.; Chung, R.; Tang, N.L.; Guo, S.; Lawton, L.-A.; Sullivan, B.J.; Wetzel, D.M.; Gunter, S.M.
Leishmania in Texas: A Contemporary One Health Scoping Review of Vectors, Reservoirs, and Human Health.

Supplementary File S1. Full Search Strategy

Overview
This document outlines the search strategy used to identify relevant literature for this scoping review. Searches were conducted in PubMed and Ovid MEDLINE for articles published between 2000 and 2024.

The following search terms were used: (“leishmania” OR “leishmaniasis”) AND (“Texas” OR “TX”). These terms were applied across keywords, titles, abstracts, and Medical Subject Headings (MeSH) terms.

PubMed
Language: English
Publication date: 2000-2024
Study type: Primary research

Search Query: (("Leishmania"[Mesh]) OR "Leishmaniasis"[Mesh] OR leishmaniasis OR leishmania) AND ("Texas"[Mesh] OR Texas”)
